# Noninfectious sternal wound inflammation after coronary artery bypass grafting in a patient with myelodysplastic syndrome: A no‐touch approach

**DOI:** 10.1111/jocs.16570

**Published:** 2022-05-06

**Authors:** Lotte ten Dam, Miarca ten Broeke, Angelique M. Poot, Martijn D. Gilbers, Frank R. Halfwerk

**Affiliations:** ^1^ Department of Cardio‐Thoracic Surgery Thoraxcentrum Twente, Medisch Spectrum Twente Enschede The Netherlands; ^2^ Department of Dermatology Medisch Spectrum Twente Enschede The Netherlands; ^3^ Department of Biomechanical Engineering, Faculty of Engineering Technology University of Twente Enschede The Netherlands

**Keywords:** cardiac surgery, myelodysplastic syndrome, pyoderma gangrenosum

## Abstract

Pyoderma gangrenosum (PG) is a rare, chronic inflammatory noninfectious dermatosis. It is associated with underlying systemic or hematological diseases such as myelodysplastic syndrome (MDS) and can be triggered after surgery. Recognition and diagnosis of PG can be difficult as it can mimic a wound infection. Misdiagnosis could lead to invasive procedures which worsen the disease and have possible disastrous aftermath. A 74‐year‐old male with a history of MDS presents with an atypical sternal wound inflammation. Diagnosis confirmed PG after skin biopsy. No surgical or invasive procedures were performed and the patient was treated on an outpatient basis with prednisolone, clobetasol cream, and cyclosporine. This case shows the importance of a rapid diagnosis of the disease. Awareness is required for the diagnosis of PG in a wound with pronounced livid borders, without improvement after antibiotic treatment or worsening after debridement. Rapid diagnosis and treatment reduce high healthcare costs, morbidity, and mortality.

## 1 INTRODUCTION

Pyoderma gangrenosum (PG) is a rare, chronic inflammatory noninfectious neutrophilic dermatosis.^1^, ^2^ Half of the cases are associated with underlying diseases. In patients younger than 65, systemic inflammatory diseases such as inflammatory bowel disease (IBD) or Morbus Crohn are most common. Underlying hematological diseases, such as myelodysplastic syndrome (MDS) or polycythemia vera are more frequently seen in patients older than 65 years.^1^, ^3^ In 20%–50% of the cases, PG is triggered by an injury, surgery, or infection.^1^, ^3^, ^4^ Incidence rates are approximated at 0.63% per 100,000.^5^

We present a case of a patient with PG following coronary artery bypass grafting (CABG) presenting 11 days postoperative. This case differs from other cases because of the multidisciplinary and noninvasive approach whereas a recent systematic review of 15 case reports showed that in 75% of PG cases, the initial diagnosis of a surgical site infection resulted in contraproductive treatments.^5^

## 2 PRESENTATION OF CASE

A 74‐year‐old male with a history of MDS after non‐Hodgkin lymphoma treated 11 years ago with splenectomy and chemotherapy was admitted with acute myocardial infarction. Coronary angiography was performed which showed three‐vessel coronary artery disease. The patient underwent urgent, uneventful revascularization with off‐pump CABG with the use of a radial artery and left internal thoracic artery. The initial patient's recovery was swift and uneventful, without clinical signs of infection, and the patient was discharged on the sixth postoperative day.

**Figure 1:**
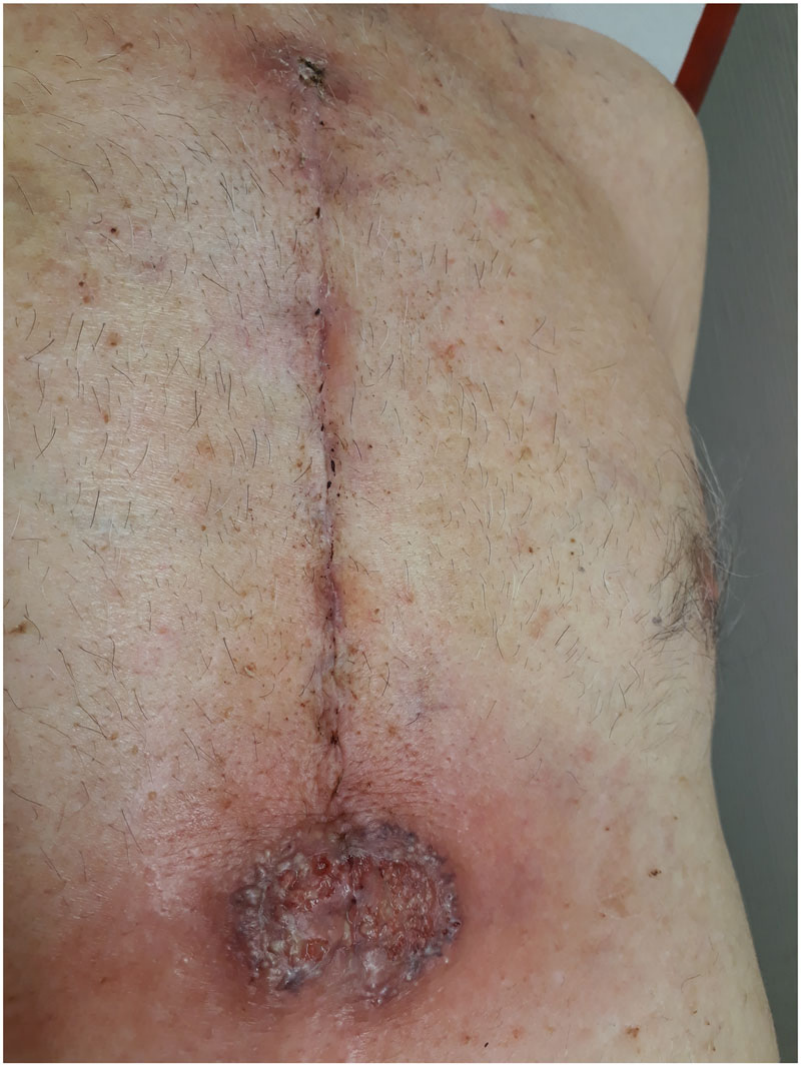
First presentation a superficial ulcer with violaceous and erythematous borders in the caudal portion of the coronary artery bypass grafting scar, 5 days after hospital discharge.

**Figure 2:**
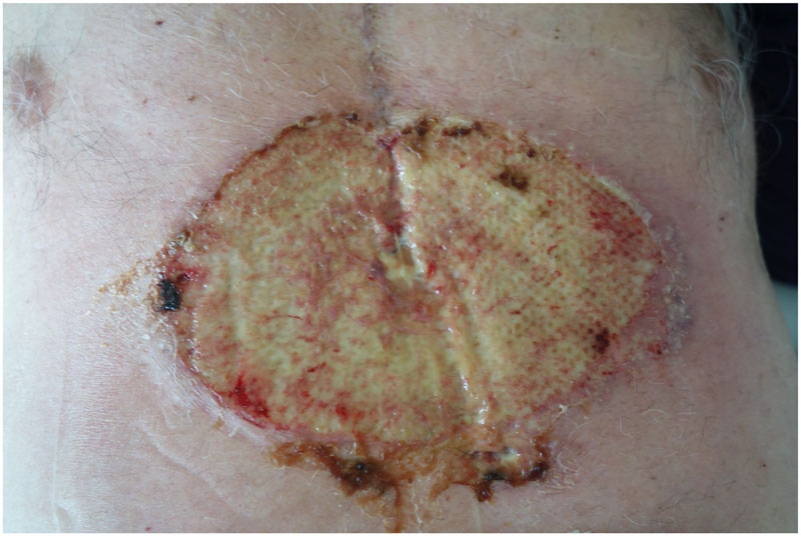
Serosanguinous drainage from the distal area of the sternal wound and rapid progression of the wound, 8 days after initial hospital discharge.

**Figure 3:**
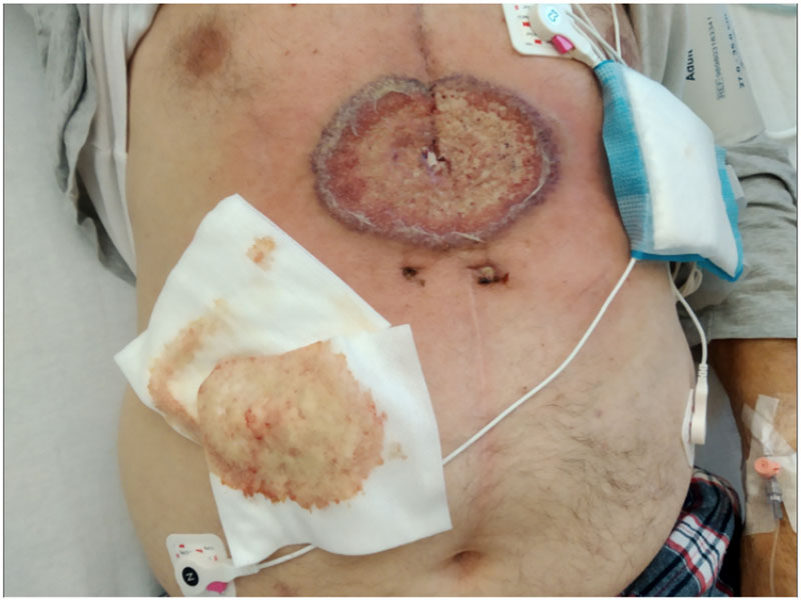
Clinical deterioration and progression of the sternal wound despite antibiotic treatment, 10 days after initial hospital discharge.

Five days after hospital discharge the patient was referred by his general practitioner because of presumed sternal wound infection. Physical examination showed a superficial circular moist ulcer with violaceous and pustular, undermined borders surrounded by erythema, located caudally in the sternal CABG scar (Figure 1). The patient was subfebrile (37.9°C), had no pain, and showed no signs of sternal instability. A superficial wound swab was taken and empiric antibiotic treatment was prescribed with amoxicillin/clavulanic acid. Three days later, the patient was admitted to the emergency department with a high fever (39.2°C), serosanguinous drainage from the caudal area of the sternal wound, and rapid progression of inflammation, with a CRP of 103 mg/L (Figure 2). Preliminary wound culture showed no bacterial growth. Antibiotic treatment was pragmatically broadened to clindamycin as a beginning deep sternal wound infection (DSWI) was suspected. The patient was discharged that same day but returned after 2 days with general malaise, high fever (40.0°C), and a painful wound with the progression of size and inflammation despite antibiotic treatment. Because of definitive negative wound cultures and an unusual, nonspecific clinical course of the presumed infection compared to a typical sternal wound infection a dermatologist was consulted (Figure 3).

**Figure 4:**
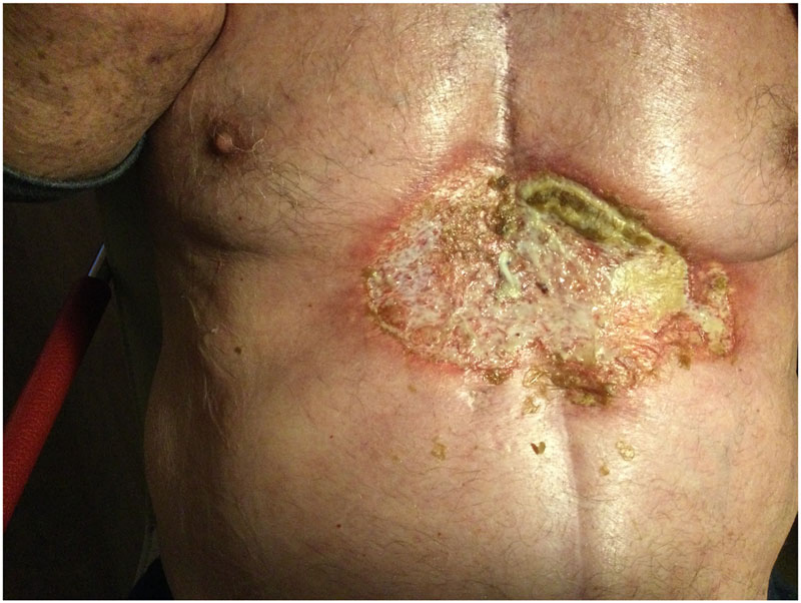
Two months after diagnosis the ulcer was almost fully cured: the wound showed marked re‐epithelialization and calm borders.

The clinical aspect of the sternal wound with prominent violaceous undermined borders strongly pointed to the diagnosis of PG. Prednisolone was started and a skin biopsy was performed which confirmed the diagnosis. Retrospectively, adding criteria such as rapid progression, absence of a relevant differential diagnosis, severe pain, and a reddish‐violaceous wound border resulted in a PARACELSUS score of more than 10 points, indicating a high likelihood of PG.^6^ Consecutively a hematologist was consulted because of history of MDS, who did not find evidence for a new lymphoma or underlying disease other than surgery as a trigger. Systemic treatment with prednisolone 1 mg/kg and cutaneous treatment with 0.5 mg/g clobetasol cream was started. The patient was discharged from the hospital after 6 days. Follow‐up took place on an outpatient basis by the dermatologist. Because of further deterioration of the wound, systemic treatment was extended with ciclosporin 5 mg/kg. After starting with prednisolone the patient developed superinfections twice. The wound culture showed *Klebsiella pneumonia* which was treated with ciprofloxacin 500 mg. He also developed painful ulcerations of the oral mucosa, which tested positive for the herpes simplex virus, and valaciclovir was prescribed. He also developed steroid‐induced diabetes mellitus which was treated with insulin and gliclazide. Two months after diagnosis the treatment with prednisolone and ciclosporin was stopped after gradually tapering down, and the ulcus was almost fully cured with calm borders (Figure 4). Wound cultures showed no bacterial growth. An overview of wound healing and deterioriation starting from first clinical presentation to complete remission is shown in a Supplementary Figure.

## 3 DISCUSSION

Surgical site infections such as superficial wound infections, DSWI, and DSWI complicated with osteomyelitis after cardiac surgery are not uncommon. With an incidence rate of 0.25%–5%,^5^, ^7^ it is of utmost importance to recognize possible underlying pathophysiological mechanisms. An erroneous diagnosis could prompt to perform a debridement which could possibly aggravate the sternal wound inflammation due to pathergy. This is an inflammatory response to, even minor, trauma or surgery that leads to the development of ulcers that may be resistant to healing.^3^ As our patient presented with unspecific signs of inflammation, it is tempting to look for the most common cause. An experienced eye can be of great importance to prevent the clinician from being led astray from the actual diagnosis and make the difference in recognizing an abnormal wound that could require a multidisciplinary approach.

Mortality rates of PG can lead up to 30% because of potential delayed diagnosis and the association between PG and underlying systemic diseases or hematological malignancies.^3^, ^8^ This underlines the importance of a rapid diagnosis and treatment. The treatment for PG should primarily focus on the underlying disease, reducing inflammation, pain management, and wound care. First‐line drugs for systemic treatment are steroids and ciclosporin.^1^, ^3^ Initial treatment of PG leading to an immunocompromised state in combination with a large wound area leads to an increased risk for bacterial superinfections. Especially in patients postsurgery with possible foreign body material, this could have potentially devastating consequences.

**Figure 5:**
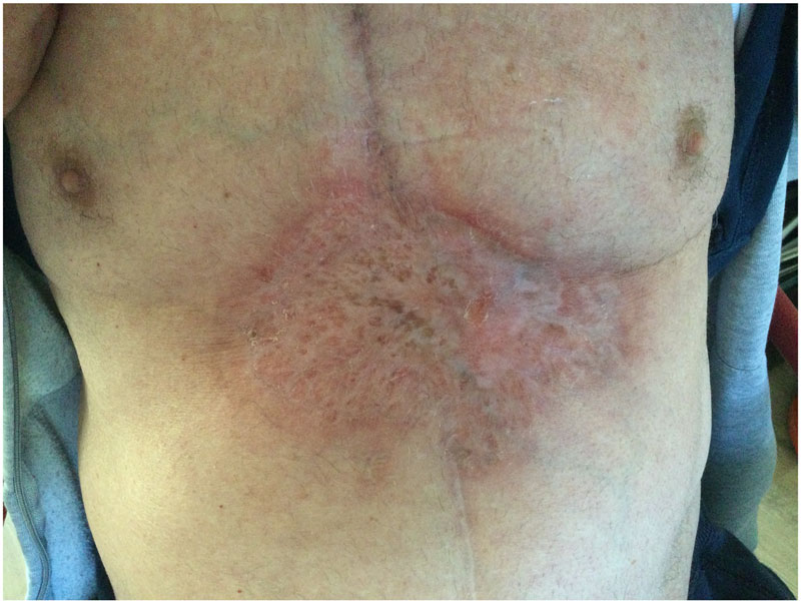
Appearance of a healed wound.

In previously reported cases of PG after cardiac surgery, treatment varied substantially.^4^, ^9^, ^10^ The majority reported a variety of surgical wound debridement which resulted in exacerbation of the inflammatory skin condition and prolonged hospital stay.^3^, ^9^ Most cases of PG with hematological malignancies as a risk factor were preceded by MDS.^1^ What makes our case unique is the adequate assessment by the nurse practitioner who performed the clinical examination and the rapid diagnosis by the dermatologist, which was made within 2 weeks after the first presentation. This has led to adequate treatment by the dermatologist and invasive treatments were avoided. Furthermore, the patient could be treated on an outpatient basis instead of a prolonged hospital stay. After a 5‐month treatment, the wound appeared healed (Figure 5).

## 4 CONCLUSION

PG is a rare complication after cardiac surgery. However, it is important to consider PG in the case of an inflamed wound that does not improve with antibiotic treatment and shows deterioration after debridement. In patients with risk factors such as for example, IBD and Morbus Crohn or hematological malignancies one should contemplate before initiating surgery and consider dermatological consultation. A multidisciplinary approach for rapid diagnosis reduces mortality, morbidity, and high costs for prolonged in‐hospital stay.

## AUTHOR CONTRIBUTIONS

Miarca ten Broeke and Lotte ten Dam were involved in data analysis/interpretation and article drafting. Martijn D. Gilbers was involved in article drafting. Angelique M. Poot was involved in patient treatment and critical revision of the article. Frank R. Halfwerk was involved in the critical revision and approval of the article, drafting supplemental figure.

## CONFLICT OF INTEREST

The authors declare no conflicts of interest.

## ETHICS STATEMENT

Written consent was given by the patient for writing this case report and the use of the photographs.

## Supporting information

Overview of wound healing and progression starting from first clinical presentation to complete remission. From left to right.
