# Supplementary figures and images for: Noninfectious sternal wound inflammation after coronary artery bypass grafting in a patient with myelodysplastic syndrome: A no‐touch approach
Source: J Card Surg. 2022 May 6;37(8):2419–22. doi: 10.1111/jocs.16570 (PMC9321867; doi:10.1111/jocs.16570)

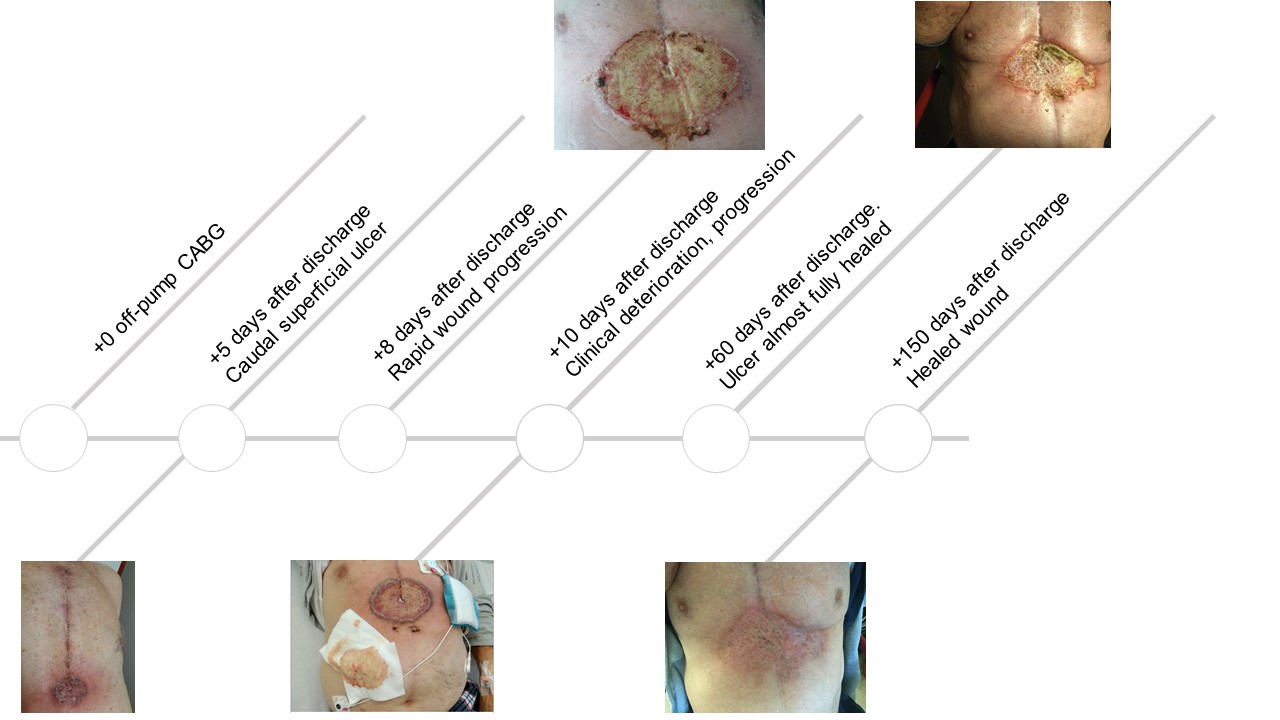

Supplement: Supplementary file 1 — Overview of wound healing and progression starting from first clinical presentation to complete remission. From left to right. [file JOCS-37-2419-s001.jpg]
